# Substantive Dimethicone-Based Mucoadhesive Coatings

**DOI:** 10.3390/ma17225590

**Published:** 2024-11-15

**Authors:** Sophie Miller, Nicole Omoto, Ryan DeCamp, Gavin Gloeb, Stephen M. Gross

**Affiliations:** 1 Department of Chemistry, College of Arts and Sciences, Creighton University, Omaha, NE 68178, USA; sophiemiller@creighton.edu (S.M.); ryandecamp@creighton.edu (R.D.); gavingloeb@creighton.edu (G.G.); 2 Department of Oral Biology, School of Dentistry, Creighton University, Omaha, NE 68178, USA; nicoleomoto@creighton.edu

**Keywords:** dimethicone, mucoadhesion, fluoride, hypersensitivity

## Abstract

It is challenging to deliver therapeutics in the oral environment due to the wet surfaces, the nature of the mucosa and the potential for saliva washout. In this study, the development of a mucoadhesive dimethicone-based oral carrier system for adhesion to the hard tissue and mucosa in the mouth was examined. This study reports the viscosity and mucoadhesion of dimethicone based polymer blends. The viscosity of the materials was measured using a rheometer. The mucoadhesion of these materials was determined as the work of adhesion and peak tack force using the tensile test method with a texture analyzer. Materials were prepared with either calcium and phosphate salts or sodium fluoride as potential therapeutics for promoting remineralization and treating dentin hypersensitivity by mechanical occlusion. Scanning electron microscopy was used to look at mineral deposition on the surface of dental hard tissue after the application of the dimethicone-based formulations. The results of this study confirm the potential for using these dimethicone-based materials as mucoadhesive therapeutic delivery systems in the oral environment.

## 1. Introduction

An ongoing challenge in oral treatments is to deliver therapeutic agents to the oral cavity with a substantive carrier. A challenge exists in maintaining a therapeutic agent in contact with a targeted absorption site. Reviews on mucoadhesive materials have been reported in the literature [1,2,3,4]. There are several reviews written on the theories of mucoadhesion that contribute to the current understanding of the phenomenon [5,6,7,8,9,10]. These include the electronic theory, adsorption theory, wetting theory, diffusion theory, fracture theory and mechanical theory [10,11,12,13,14,15]. The concepts of attraction behind these theories after surface wetting rely on attraction based on covalent bonds, non-covalent interactions (e.g., hydrogen bonding, van der Waals forces), interpenetration and chain entanglement and hydrophobic interactions. Depending upon the mucoadhesive used, a combination of these forces is used to explain the mechanism of adhesion for a specific material.

Due to the fact that many mucoadhesive polymers bind via multiple mechanisms, these carrier systems are often also classified by the source of the material. These classifications include natural, semisynthetic and synthetic mucoadhesives [16,17,18]. Some common examples of the natural materials include gelatine, carrageenan, pectin, starch, guar gum, xanthan gum, gallan gum and hyaluronic acid. Some of the common semisynthetic materials include a variety of derivatized celluloses and chitosan. For synthetic materials, PEG, PVA, PAA, PVP and thiolated polymers are reported in the literature and are found in commercial products.

Dimethicones are generally regarded as safe (GRAS) and have been used for decades in numerous medical and cosmetic applications [19,20,21]. Dimethicone itself is the active ingredient in some orally delivered anti-acid and anti-gas medications acting to reduce the surface tension of gas bubbles. Dimethicone is typically used as an excipient in numerous oral and dermal healthcare products. The exceptional properties of dimethicone stem from the partial ionic character and high bond energy of the siloxane bond, the significant backbone flexibility and the low intermolecular forces between the compact pendant methyl groups [22]. For dermal applications, dimethicone is typically added from 1 to 30 weight percentage of a formulation to improve substantivity and act as a skin protectant [23]. When used an additive, dimethicone is known to impart lubricity to a formulation and improve other handling characteristics such as spreadability and tackiness [24]. Dimethicones have also been modified with aqueous systems for use in emulsions to deliver therapeutics [25,26]. Dimethicone has also been incorporated into pressure sensitive adhesives as a component for transdermal drug delivery [27,28,29,30].

In this study, the possibility of using a dimethicone polymer blend as the primary oral carrier system (not as an excipient or active ingredient or part of a more complex delivery system) is examined. While various mucoadhesive polymer systems in the oral environment currently exist, some challenges or limitations of these oral delivery systems provide an opportunity for a new carrier system. Some of the current challenges include limited substantivity. Many existing products that are applied to the surface of the soft and hard tissue slide quickly from the applied surface due to saliva washout or are mechanically removed by simply pushing with the tongue. Some systems use solvents, which leads to challenges in manufacturing and product stability and poor patient experience due to the flashing of the solvents once in contact with the mucosa or hard tissue. Another challenge is delivering incompatible components in the same moiety. For example, if a remineralizing or hypersensitivity treatment would benefit from the inclusion of both calcium and phosphate salts, the use of an aqueous mucoadhesive system would lead to the precipitation of insoluble calcium phosphates in the formulation itself and not on the tooth, leading to formulation stability and packaging issues.

The use of a dimethicone blend can address these challenges. The use of dimethicone as the carrier can act as a solvent-free system for a patient with outstanding aesthetic and organoleptic properties (e.g., a lubricious mouth feel) while providing a potential substantive carrier system with oral (and potentially dermal) applications. The use of dimethicone without a solvent would greatly simplify manufacturing and packaging issues associated with solvents. Dimethicone blended with salts that would otherwise precipitate in aqueous systems would remain in salt forms that would readily dissolve once applied in the oral environment, rather than in the formulation itself. A blend of dimethicones wherein one dimethicone with a low viscosity (but non-volatile) is used to impart spreadability in conjunction with an extremely high viscosity dimethicone to impart substantivity can be used as a carrier system. This two part dimethicone system can be used in conjunction with a small amount of an edible, food-grade wax as a potential carrier system with an enhanced mucoadhesion capable of releasing active ingredients.

Dentin hypersensitivity can result in pain that impacts a patient’s ability to eat, drink, talk and perform daily oral hygiene. The toothache associated with dentin hypersensitivity originates from the exposure of dentin to a variety of external stimuli [31,32,33]. Brannstrom’s hydrodynamic theory provides the most common explanation for dentin hypersensitivity and provides an explanation for why occlusion of the dentin tubules is considered a treatment for the condition [34,35,36,37,38]. Dentin tubules exposed at the enamel cementum junction or due to wear or caries in the oral environment are typical treatment sites for desensitization. Typically, gels or toothpastes are applied to the effected tooth surfaces, but the effects of these treatments can be short-lived due to the ease of removal from the surface by friction, saliva or acid or the transient relief provided by potassium ions.

This study looked to explore the use of an improved mucoadhesive material capable of occluding exposed dentin tubules that could benefit patients suffering from dentin hypersensitivity. In this work, the use of a blend of dimethicones with a small amount of an edible wax and calcium and phosphate salts or fluoride containing salts used in the remineralization of enamel or dentin was reported. The mucoadhesive properties in terms of peak tack force and work of adhesion were studied along with the viscosity of these materials. The dimethicone blend was spread across the surface of teeth with an applicator brush. Scanning electron microscopy was used to determine whether the dimethicone could occlude exposed tubules and to visualize mineral deposition on the surface of the teeth from added calcium and phosphate or fluoride sources.

## 2. Materials and Methods

*Materials Used.* In total, 12,500 and 2,500,000 cP dimethicone was obtained from Nusil (Carpinteria, CA, USA). Edible wax was obtained from Stakich (Troy, MI, USA). Artificial saliva was obtained from Pickering Laboratories (Mountain View, CA, USA). Calcium chloride, potassium phosphate dibasic and sodium fluoride was obtained from Fisher Scientific (Waltham, MA, USA).

*Formulations.* In this study, dimethicone of two different viscosities were mixed using a Flacktek speedmixer (Landrum, SC, USA). A series of formulations were prepared by mixing 12,500 cP dimethicone, 2,500,000 cP dimethicone and edible wax. Calcium chloride, potassium phosphate or sodium fluoride was added to the polymer blend. Blends of just the two dimethicones with up to 30 *w*/*w*% 2,500,000 cP were prepared for viscosity measurements. Four formulations with added wax and salts were evaluated in this study, and the compositions are reported in Table 1. The formulations were stored in a Norlake Environmental Chamber (Hudson, WI, USA) at 45 °C and 65% humidity for 6 months and exhibited no phase separation, indicating that the formulations would have a shelf-life stability of at least 2 years at room temperature.

*Viscosity*. Viscosity measurements were determined using a DVT3 Brookfield Engineering Rheometer (Middleboro, MA, USA) in a small sample chamber using a SC4-18 spindle. Colloids were sheared at a rate of 1 rpm (1.32 s^−1^) for 90 s and then 10 rpm (13.2 s^−1^) for 90 more seconds.

*Mucoadhesion*. The mucoadhesion of dimethicone-based formulations were measured using the tensile test method, as described in the literature [39,40,41,42,43]. A Stable Micro Systems TA.XT Express instrument (Surrey, UK) was used with a TA-4 probe at room temperature (23 ± 1 °C). The Peak Adhesive Force and the Total Work of Adhesion were measured (n = 6). The force required to remove the test probe from the sample determined the Peak Adhesive Force. This was measured by recording the peak maximum force. The Total Work of Adhesion was determined by how much work was required to pull the probe out of the sample. This was measured by integrating the area between the force–time curve and the x-axis while removing the probe from the sample. By the nature of the technique, the larger negative value correlated to a larger peak tack force and work of adhesion. A one-way ANOVA test was completed to determine if the data means were statistically significant, resulting in a *p*-value of less than 0.05. A multiple comparison of each mean (Tukey’s test) was then completed at a 95% confidence interval, and groupings were determined by a lettered superscript on the formulation group in thedata reported for the peak tack force and the work of adhesion.

*Dentin Occlusion and Scanning Electron Microscopy*. Human molar specimens were polished with 320 grit polish paper at 70 rpm until the dentin was visible. The teeth were then soaked in 1 M perchloric acid for 10 s to reveal a clean exposed dentin surface. The specimens were treated with dimethicone based formulations on exposed dentin tubules. The formulations were brushed on with a typical varnish applicator. The specimens were then soaked in artificial saliva for 24 h. The dimethicone treatment was removed from the surface by soaking with hexane (the dimethicone could not readily be removed by rinsing with water due to the mucoadhesion to the dentin surface) for 30 min. The top-down images of the dentin surfaces were imaged on a Hitachi TM3000 scanning electron microscope (SEM) (Tokyo, Japan). Each specimen was sputter coated with gold prior to imaging.

## 3. Results

**Figure 1:**
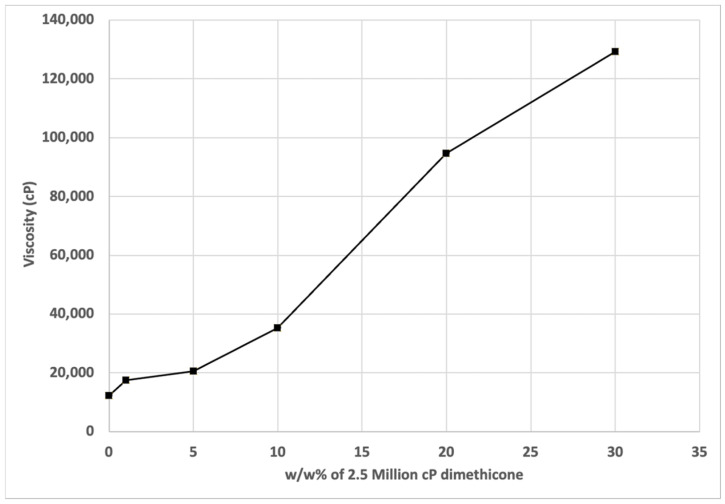
Viscosity of 12,500 cP and 2.5 million cP dimethicone blends as a function of *w*/*w*% of the 2.5 million cP component in the blend. The viscosity was measured at a shear rate of 1.32 s^−1^.

Dimethicone-based blends containing ionic elements that promote remineralization were prepared as potential dentin hypersensitivity treatments. Initially, the viscosity of the pure polymer blends with up to 30 *w*/*w*% 2.5 million cP dimethicone were measured to determine the handling characteristics of these materials. As seen in Figure 1, there was a significant increase in the viscosity going from 5 to 10 *w*/*w*% 2.5 million cP dimethicone. Therefore, the formulations with added wax and salts focused on formulations with 5 *w*/*w*% 2.5 million dimethicone. Formulations with 5 *w*/*w*% of the 2.5 million cP dimethicone were very easy to spread with a standard varnish applicator brush. While the dimethicone blend could still spread with 10 *w*/*w*% of the 2.5 million cP dimethicone, it would require significantly more effort, which would likely turn off potential users of a treatment.

**Figure 2:**
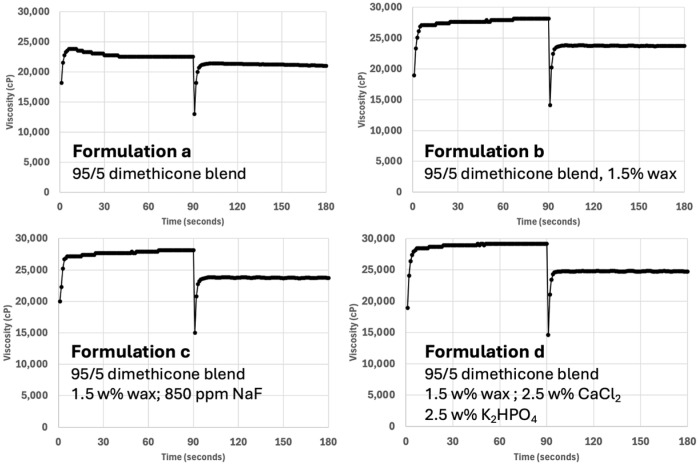
The viscosity as a function of time at shear rates of 1.32 s^−1^ for 90 s and 13.2 s^−1^ held for 90 s at each shear rate. Formulation (a) represents the 95/5 blend of 12,500 cP dimethicone/2.5 million cP dimethicone. Formulation (b) represents the addition of 1.5 *w*/*w*% of wax to the 95/5 dimethicone blend. Formulation (c) represents the addition of 850 ppm NaF to the 95/5 dimethicone blend with added wax. Formulation (d) represents the addition of 2.5 *w*/*w*% CaCl_2_ and 2.5 *w*/*w*% K_2_HPO_4_ to the 95/5 dimethicone blend with added wax.

Figure 2 reports the viscosity of the blends with (a) a 95/5 mixture of 12,500 cP with 2,500,000 cP dimethicone, (b) a 95/5 dimethicone blend with 1.5 *w*/*w*/% wax, (c) a 95/5 dimethicone blend with wax and 850 ppm sodium fluoride and (d) a 95/5 dimethicone blend with wax and 5 *w*/*w*% calcium chloride and potassium phosphate dibasic salt. As seen in Figure 2, all four formulations shear-thinned with an increase in shear rate from 1.32 s^−1^ to 13.2 s^−1^. This pseudoplastic behavior was more pronounced in formulations (b–d), which included the wax. The addition of 850 ppm sodium fluoride had no discernible effect on the viscosity between graphs for formulations (b) and (c). At the lowest shear rate, the formulations were non-Newtonian as a function of time. The pure dimethicone blend exhibited thixotropic behavior as a function of time, as seen in formulation (a). However, formulations (b–d) behaved rheopectically with an increase in viscosity over time. At the higher shear rate of 13.2 s^−1^, all four formulations essentially behaved in a Newtonian manner with the viscosity remaining constant over time. While a small amount of wax slightly increased the viscosity, the formulation still spread easily across a tooth with little effort. Significant increases in wax content, similar to the addition of high-viscosity dimethicone, rapidly made the material more difficult to spread easily on the surface of the tooth.

**Figure 3:**
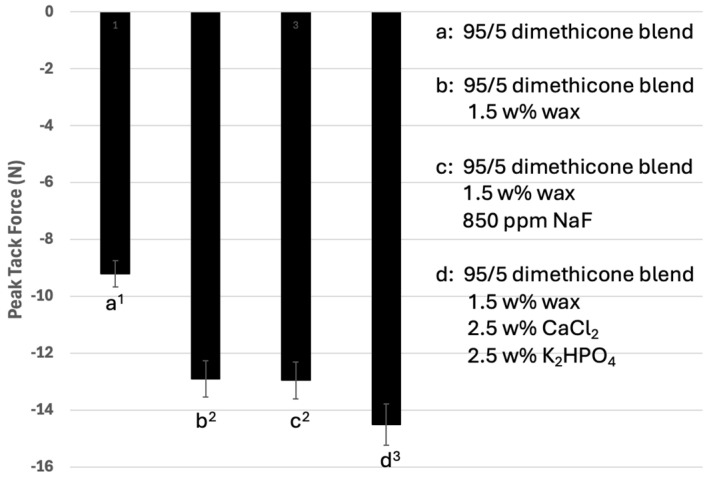
The peak tack force of the formulations tested by the tensile test method with a texture analyzer. Formulation (a) represents a 95/5 blend of 12,500 cP dimethicone/2.5 million cP dimethicone. Formulation (b) represents the addition of 1.5 *w*/*w*% of wax to the 95/5 dimethicone blend. Formulation (c) represents the addition of 850 ppm NaF to the 95/5 dimethicone blend with added wax. Formulation (d) represents the addition of 2.5 *w*/*w*% CaCl_2_ and 2.5 *w*/*w*% K_2_HPO_4_ to the 95/5 dimethicone blend with added wax.

The mucoadhesion of the blends was reported in terms of the peak tack force and the work of adhesion of the formulations. Using the tensile test method on a texture analyzer, Figure 3 reports the peak tack force of the blends with (a) a 95/5 mixture of 12,500 cP with 2,500,000 cP dimethicone, (b) a 95/5 dimethicone blend with 1.5 *w*/*w*/% wax, (c) a 95/5 dimethicone blend with wax and 850 ppm NaF and (d) a 95/5 dimethicone blend with wax and 2.5 *w*/*w*% calcium chloride and 2.5 *w*/*w*% potassium phosphate dibasic salt.

**Figure 4:**
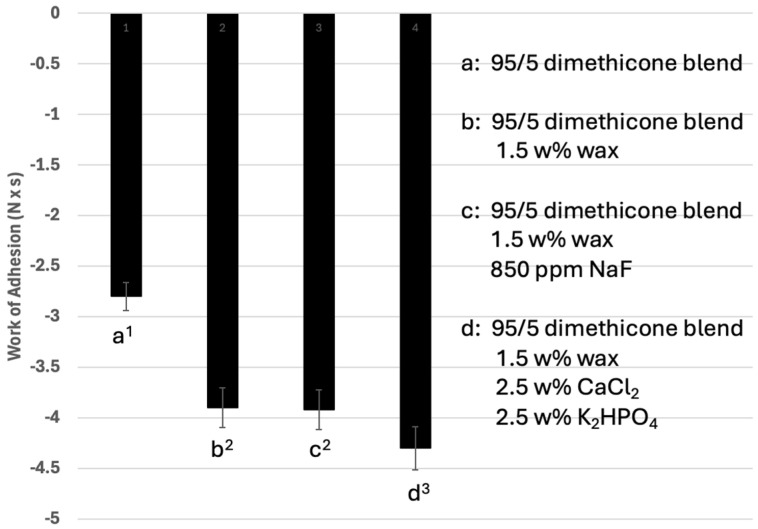
The work of adhesion of the formulations tested by the tensile test method with a texture analyzer. Formulation (a) represents a 95/5 blend of 12,500 cP dimethicone/2.5 million cP dimethicone. Formulation (b) represents the addition of 1.5 *w*/*w*% of wax to the 95/5 dimethicone blend. Formulation (c) represents the addition of 850 ppm NaF to the 95/5 dimethicone blend with added wax. Formulation (d) represents the addition of 2.5 *w*/*w*% CaCl_2_ and 2.5 *w*/*w*% K_2_HPO_4_ to the 95/5 dimethicone blend with added wax.

Figure 4 reports the work of adhesion of the blends with (a) a 95/5 mixture of 12,500 cP with 2,500,000 cP dimethicone, (b) a 95/5 dimethicone blend with 1.5 *w*/*w*% wax, (c) a 95/5 dimethicone blend with wax and 850 ppm NaF and (d) a 95/5 dimethicone blend with wax and 2.5 *w*/*w*% calcium chloride and 2.5 *w*/*w*% potassium phosphate dibasic salt.

As seen in Figure 3 and Figure 4, the peak tack force of the plain 95/5 dimethicone blend (formulation (a)) was −9.2 ± 0.8 N and the work of adhesion was −2.8 ± 0.23 N·s. When the wax was added in formulation (b), there was a significant increase in the peak tack force (−12.9 ± 1.1 N) and work of adhesion (−3.9 ± 0.35 N·s) compared to the dimethicone blend. The addition of 850 ppm sodium fluoride to formulation (c) did not result in a significant change in the peak tack force (−12.9 ± 0.9 N) or the work of adhesion (−3.9 ± 0.3 N·s). The addition of 2.5 *w*/*w*% calcium and 2.5 *w*/*w*% phosphate salts to formulation (d) resulted in a significant increase in the peak tack force (−14.5 ± 1.0 N) and work of adhesion (−4.3 ± 0.3 N·s).

**Figure 5:**
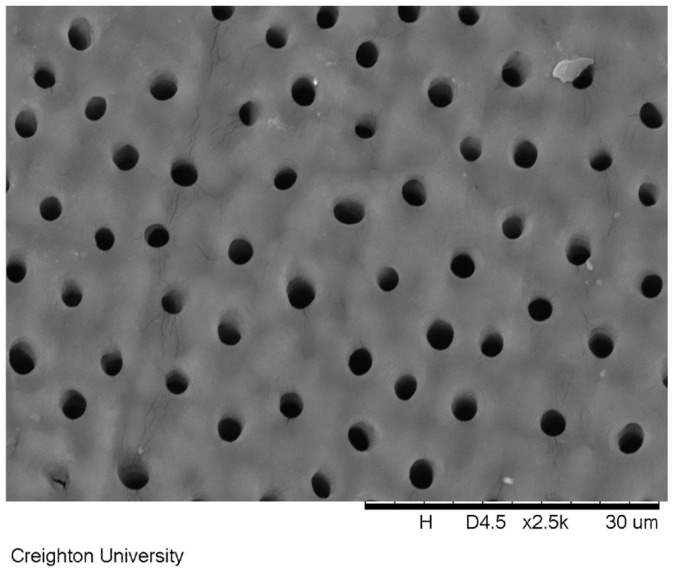
A scanning electron microscope image of the exposed dentin surface prepared for use in this study. The exposed tubules are clearly visible in the image.

Human tooth specimens with exposed dentin tubules were treated with the dimethicone blends containing either 2.5 *w*/*w*% of calcium chloride and 2.5 *w*/*w*% potassium phosphate dibasic salts or 850 ppm NaF salt with the use of a standard varnish applicator brush. After soaking in artificial saliva and the subsequent removal of the formulation, the surfaces of the exposed dentin were imaged by scanning electron microscopy. Figure 5 shows the untreated dentin surface. The exposed dentin tubules can be clearly observed in the image.

**Figure 6:**
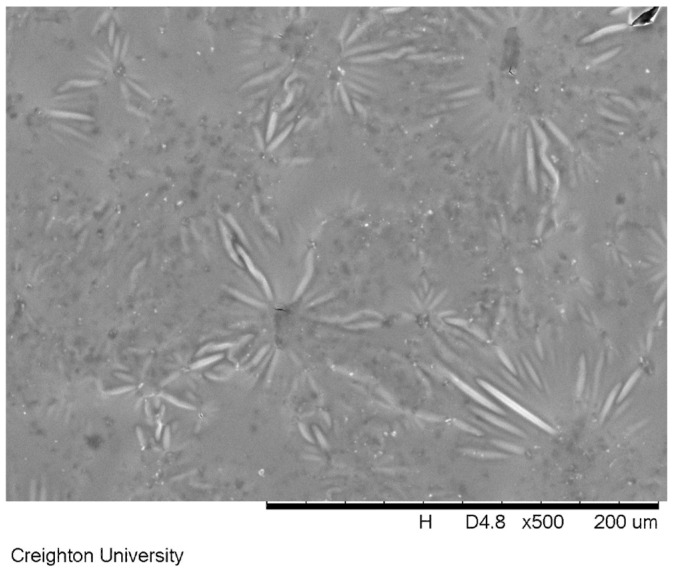
A scanning electron microscope image of the exposed dentin surface treated with a formulation containing dimethicone (95/5 of 12,500/2.5 million cP), wax and 850 ppm of sodium fluoride after soaking in artificial saliva for 24 h. The dentin tubules are completely occluded in the image.

A dentin surface coated with formulation (c) and soaked in artificial saliva for 24 h is shown in Figure 6. The dimethicone blend remained adhered to the exposed dentin surface, as evidenced by the occluded tubules in the scanning electron microscope image.

**Figure 7:**
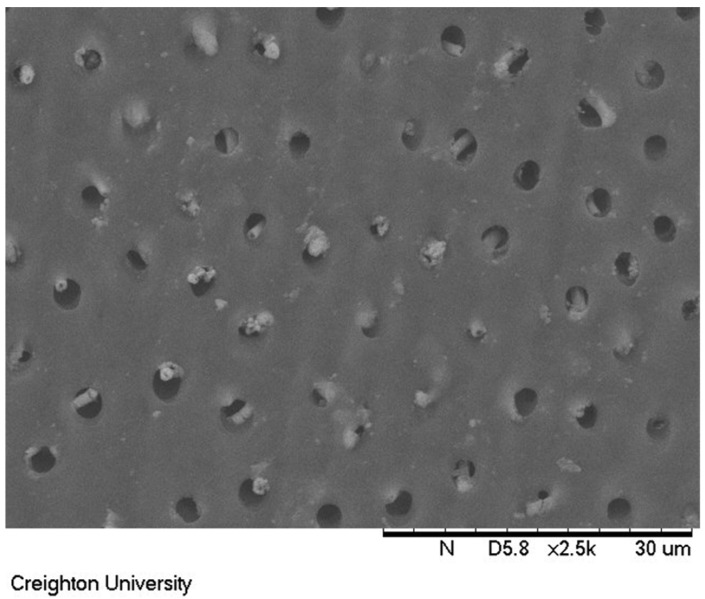
A scanning electron microscope image of an exposed dentin surface after removing the dimethicone blend with 850 ppm sodium fluoride treatment after soaking in artificial saliva for 24 h.

The surface treated with formulation (c) containing 850 ppm sodium fluoride is shown in Figure 7. This sample was soaked in artificial saliva for 24 h and had the dimethicone blend removed. Columnar-shaped precipitates can be observed extending from the top of exposed dentin tubules in this image.

**Figure 8:**
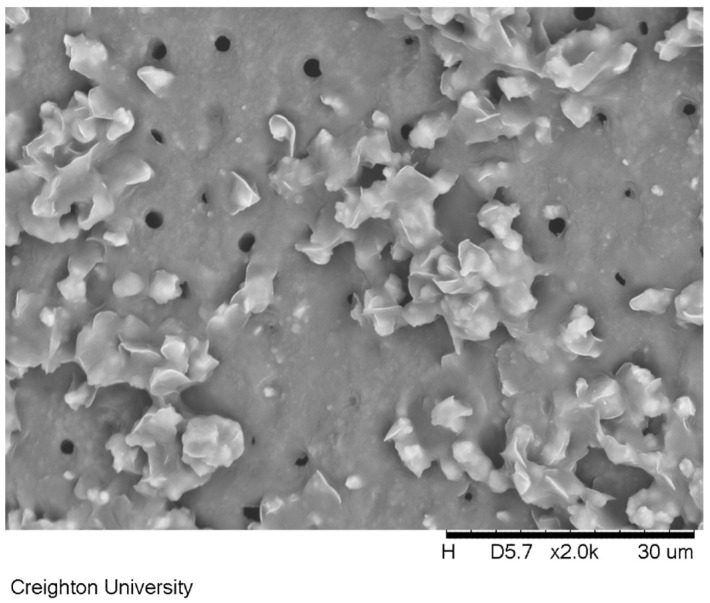
A scanning electron microscope image of an exposed dentin surface after removing the calcium chloride and potassium phosphate dibasic dimethicone blend after soaking in artificial saliva for 24 h.

Figure 8 shows the dentin surface treated with formulation (d) containing calcium and phosphate salts. This specimen was soaked in artificial saliva for 24 h after the application of the formulation. The removal of the dimethicone formulation revealed precipitates extending from the dentin surface.

## 4. Discussion

The occlusion of dentin tubules is regarded as an accepted treatment of dentin hypersensitivity. While toothpaste and gels provide temporary relief from the symptoms of dentin hypersensitivity, patients could potentially feel longer-term relief from the symptoms if a treatment with greater substantivity could be applied to the exposed dentin surface with the ability to not only provide a barrier between the hard tissue surface and the pulp chamber but also deposit minerals over or in the exposed tubules to provide longer-term relief.

In order to create a substantive coating to the surface of the dentin, dimethicones with two significantly different viscosities were initially blended. The lower viscosity dimethicone (12,500 cP) provided the ability to easily spread the formulation over the dentin surface. The extremely high viscosity dimethicone (2,500,000 cP) on its own did not spread over a surface. However, when blended and well entangled in a homogenous mixture with the lower viscosity polymer, the high viscosity polymer readily spread across the surface of the exposed dentin and was believed to add significant substantivity of the blend as it greatly increased the peak tack force and work of adhesion of the coating. The addition of a small amount of wax enhanced the mucoadhesive properties of these blends.

For the purpose of remineralizing the surface of dental hard tissue, the use of calcium, phosphate and fluoride salts is well established in dental materials. Therefore, two different approaches were explored in this study to remineralize the exposed dentin surface. In one approach, the use of water (saliva)-soluble calcium-containing and phosphate-containing salts were added to the dimethicone blend. If an aqueous carrier for the salts was used, the calcium-soluble and phosphate-soluble salts would dissolve in the formulation and precipitate insoluble calcium phosphate minerals. However, a non-aqueous carrier such as dimethicone could be used to carry the saliva-soluble salts to the surface of the dentin. Then, when in contact with saliva, the salts could dissolve at the target location of the dentin surface and subsequently precipitate on the surface of the dentin, rather than in the formulation itself had the carrier been aqueous-based. The addition of an edible wax that melts and disperses through the dimethicone blend greatly enhanced the deposition of the minerals on the surface of the dentin without inhibiting the spreadability on the surface.

Figure 1 reports the viscosity of dimethicone blends with up to 30 *w*/*w*% of the 2,500,000 cP dimethicone. The addition of 1–5 *w*/*w*% of the high-molecular dimethicone increased the viscosity of the blend to approximately 17,500 and 23,000 cP, respectively. These blends were very easy to spread with a common applicator brush onto a tooth surface. Therefore, the salt-containing formulations were prepared using 5 *w*/*w*% of the high-viscosity dimethicone. Figure 2 depicts the viscosity of the dimethicone based formulations examined in this study that included the addition of wax and salts. As expected, the addition of 5 *w*/*w*% of the 2,500,000 cP dimethicone increased the viscosity of the 12,500 cP polymer initially to 23,800 cP, eventually thinning to 22,500 cP at 1.32 s^−1^. As expected, the polymer blend shear-thinned with an increase in the shear rate. The inclusion of the wax significantly increased the viscosity of the blend initially to 27,300 cP, which thickened over time to 29,200 cP. This could potentially be explained by the greater intermolecular forces created by the dispersed wax, in addition to the wax acting as a dispersed particulate, causing a greater amount of energy to be required to reorganize around the spindle. Either way, the rheopecty could have originated from increased structure in the fluid. The addition of 850 ppm sodium fluoride to the formulation had no discernible effect on the formulation viscosity. However, the addition of 2.5 *w*/*w*% calcium and 2.5 *w*/*w*% phosphate salts led to a slight increase in the dimethicone/wax blend, likely due to increased particulate dispersal in the colloid.

Figure 3 depicts the peak tack force values of the formulations, as determined by the tensile test method using a texture analyzer. The peak tack force is a measure of how quickly an adhesive bond is formed when two surfaces are brought together with light pressure and brief contact. In this experiment, a test probe was brought into contact with the formulation at a fixed speed, contact pressure and contact time. The peak tack force represents the maximum force required to break the resultant bond. The more negative value represents greater adhesion in this method. Formulation (a) (dimethicones only) has a very large fraction of low-viscosity dimethicone that allows for easy spreading and, therefore, wetting of the formulation onto the surface. The low-viscosity fraction blends homogenously with the high-viscosity fraction, allowing for the entanglement of the dimethicones of significantly different molecular weights. Once spread on the surface, the high-viscosity fraction allows for substantive adhesion to the surface. For the dimethicone component of the formulation, additional mucoadhesion potentially comes from the partial ionic nature of the silicon–oxygen bond. The inclusion of the wax increases the dimethicone mucoadhesive properties, potentially due to increased molecular interactions from the presence of ester, carboxylic acid and alcohol functional groups in the structure. These functional groups can lead to increased dipole–dipole interactions and hydrogen bonding. The inclusion of 850 ppm NaF did not significantly effect the peak tack force of the formulation, presumably due to the very small quantity present in the mixture. The inclusion of 5 *w*/*w*% calcium and phosphate salts did result in a significant increase in the measured peak tack force of the formulation. This is potentially due to greater adsorption forces from more dipole and electronic interactions. Figure 4 depicts the work of adhesion of these blends. The work of adhesion correlates to the adhesive or cohesive strength of a formulation. Just like the peak tack force experiment, a test probe was brought into contact with a formulation at a fixed speed, contact pressure and contact time. The work of adhesion was calculated by measuring the area under the force–time curve. In this method, the more negative value represents the greater work of adhesion. In this study, the general trends of work of adhesion followed that of the peak tack force.

One widely accepted approach to alleviate pain from dentin hypersensitivity can be accomplished by occluding exposed dentin tubules. Figure 5 shows an untreated dentin surface with exposed tubules. Temporary relief can be gained by applying a gel-like material to the teeth to occlude these tubules. With commercially available treatments, this approach typically offers limited relief due to the lack of substantivity of the formulation to the hard tissue. However, the peak tack force and work of adhesion of the dimethicone blends is significantly higher than common OTC oral treatments. Even after soaking in artificial saliva for 24 h, the dimethicone formulations remains intact on the surface of the dentin. An example of the dimethicone formulation occluding the surface exposed tubules is seen in Figure 6 for the sample containing 850 ppm sodium fluoride (formulation c). Figure 6 demonstrates that after 24 h of soaking in artificial saliva, the dimethicone formula still remained adhered to the dentinal surface, occluding all of the tubules.

Exposed dentin treated with the 850 ppm sodium fluoride formulation that was soaked in artificial saliva for 24 h and subsequently removed is shown in Figure 7. The surface of the exposed dentin after treatment with 850 ppm sodium fluoride revealed precipitated mineral in the tubules. The cylindrical deposits of mineral was found not only on the surface of the exposed dentin but also within the actual tubules. This provides evidence that the dimethicone was not only covering the exposed dentin tubules but was also infiltrating the top of the exposed tubules with the micronized sodium fluoride. Subsequent exposure to artificial saliva potentially provided adventitious calcium ions to precipitate released fluoride ions directly in the tubule providing for a possibly more significant treatment.

Figure 8, which depicts the dentin surface treated with a formulation with calcium chloride and potassium phosphate dibasic salts after the removal of the carrier shows a surface with precipitated calcium phosphate mineral. This result demonstrates the potential use of the dimethicone to carry what would otherwise be incompatible ingredients for an aqueous-based treatment to the surface of the enamel. When given enough time from the use of a substantive coating, the saliva can dissolve the two water-soluble salts right at the surface of the dentin and precipitate the insoluble calcium phosphate mineral directly on the surface of the dentin. EDS used in conjunction with the SEM verified that the deposit consisted of calcium, phosphorus and oxygen atoms in the deposit. A more significant study is part of characterization efforts due to the challenge of quantifying the calcium and phosphate deposits on a surface of dentin. While the application of a dimethicone coating should provide significant temporary relief of dentin hypersensitivity due to the mechanical barrier it creates a coating on the surface of the dentin; this is not likely to have a long-term effect. However, the potential to occlude tubules with precipitated calcium phosphates grown from the surface of the exposed dentin has the potential to offer more long-term relief to a patient. The repeated application of this formulation could result in a more significant deposition of minerals that could occlude exposed tubules.

## 5. Conclusions

There is a need for more substantive coatings in the oral environment to both the hard and soft tissue. The use of two dimethicones, wherein one allows for the easy handling and spreading of a therapeutic formulation, while the other provides a substantive mucoadhesive coating, is a promising approach for the improved treatment of many conditions. These formulations demonstrated significant peak tack force and work of adhesion. SEM demonstrated the potential use of the dimethicone formulation to carry remineralizing agents to the surface of exposed dentin, which can be applied to a number of dental applications and beyond.

## Figures and Tables

**Table 1:** The composition of the mixtures evaluated in this study.

Formulation	12,500 cP Dimethicone (*w*/*w*%)	2.5 Million cP Dimethicone (*w*/*w*%)	Wax (*w*/*w*%)	Calcium Chloride (*w*/*w*%)	Potassium Phosphate Dibasic (*w*/*w*%)	Sodium Fluoride (ppm)
a	95	5	0	0	0	0
b	93.5	5	1.5	0	0	0
c	93.5	5	1.5	0	0	850
d	89	4.5	1.5	2.5	2.5	0

## Data Availability

The original contributions presented in the study are included in the article, further inquiries can be directed to the corresponding author.
